# Waterpipe Tobacco Smoke Inhalation Triggers Thrombogenicity, Cardiac Inflammation and Oxidative Stress in Mice: Effects of Flavouring

**DOI:** 10.3390/ijms21041291

**Published:** 2020-02-14

**Authors:** Abderrahim Nemmar, Suhail Al-Salam, Sumaya Beegam, Priya Yuvaraju, Nur Elena Zaaba, Javed Yasin, Badreldin H. Ali

**Affiliations:** 1 Department of Physiology, College of Medicine and Health Sciences, United Arab Emirates University, P.O. Box 17666 Al Ain, UAE; sumayab@uaeu.ac.ae (S.B.); priyay@uaeu.ac.ae (P.Y.); elenazaaba@uaeu.ac.ae (N.E.Z.); 2 Zayed Center for Health Sciences, United Arab Emirates University, P.O. Box 17666 Al Ain, UAE; 3 Department of Pathology, College of Medicine and Health Sciences, United Arab Emirates University, P.O. Box 17666 Al Ain, UAE; suhaila@uaeu.ac.ae; 4 Department of Internal Medicine, College of Medicine and Health Sciences, United Arab Emirates University, P.O. Box 17666 Al Ain, UAE; javed.yasin@uaeu.ac.ae; 5 Department of Pharmacology and Clinical Pharmacy, College of Medicine & Health Sciences, Sultan Qaboos University, P.O. Box 35, Muscat 123, Al-Khod, Oman; alibadreldin@hotmail.com

**Keywords:** waterpipe smoke, nose-only exposure, flavouring, heart, oxidative stress, inflammation, thrombosis

## Abstract

The consumption of water-pipe smoking (WPS) has been promoted by the use of flavoured tobacco. However, little is known about the impact of flavouring on the cardiovascular toxicity induced by WPS inhalation. Here, we compared the cardiovascular effects and underlying mechanism of actions of plain (P) (unflavoured) versus apple-flavoured (AF) WPS (30 minutes/day, 5 days/week for 1 month) in mice. Control mice were exposed to air. Both P- and AF-WPS inhalation induced an increase in systolic blood pressure, thrombogenicity and plasma concentration of fibrinogen and von Willebrand factor. In heart homogenates, AF-WPS inhalation caused an increase of 8-isoprostane and a decrease in the levels of reduced glutathione (GSH) and superoxide dismutase (SOD). Nevertheless, P-WPS decreased only the activity of SOD. The concentrations of tumour necrosis factor α and interleukin 1β were increased only in heart homogenates of mice exposed to AF-WPS. Although both P- and AF-WPS increased the concentration of troponin I in heart homogenates and induced DNA damage, the concentration of cleaved caspase 3 was only increased in mice exposed to AF-WPS. Immunohistochemical analysis of the hearts showed that both P- and AF- WPS inhalation decreased the expression of SOD. Moreover, the expression of nuclear factor erythroid-derived 2-like 2 at nuclear level in the heart was higher in both AF-WPS and P-WPS compared with control group, and the effect observed in AF-WPS group was more significant than that seen in P-WPS group. Likewise, the concentration of heme oxygenase-1 was significantly increased in both P-WPS and AF-WPS groups compared with control group, and the effect seen in AF-group was higher than that observed in P-WPS group. In conclusion, our findings showed that both P- and AF-WPS induce thrombogenicity and cardiac injury, and that this toxicity is potentiated by the presence of flavouring.

## 1. Introduction

Waterpipe smoking (WPS) has developed rapidly into a global tobacco epidemic that has received wide acceptance, predominantly among the youth. In the latter, a prevalence of WPS use of 2.5% to 37.2% has been reported in Eastern Mediterranean countries, whereas in European countries and the USA, the prevalence was 2.2% to 22.7% and 1.0% to 11.4%, respectively [1]. Moreover, a recent study from the USA found that 18.2 % of adults (18–24 years) report current use of WPS compared with 19.6% who smoke cigarettes and 8.9% who report the use e-cigarette [2]. 

Studies assessing the similarity between cigarette smoking (CS) and WPS indicated that one daily session of WPS equates smoking of 10 cigarettes per day [3]. Moreover, it has been reported that the adverse pulmonary and systemic effects of WPS are comparable or even more marked than those of CS [4,5,6]. Several factors have been quoted to explain the significant increase in WPS use, including the misperception that it is less addictive and less harmful than CS. In fact, several WPS users indicated that one of the reasons for their preference of WPS over CS is that they are confident of their capacity to abandon WPS when they want and that the water filters out the toxins [7,8]. Moreover, it has been reported that the use of flavoured fruit scent waterpipe tobacco to override the formerly intolerable aroma has increased its attractiveness to first-time users, particularly teenagers [6,9,10]. In fact, youths cite the pleasant taste and smell of WPS to explain the reasons for their use of WPS [6,9,10]. However, little is known about the health effects of the flavouring used in WPS. 

Experimental and clinical studies have demonstrated that the consumption of tobacco products having flavouring and flavour enhancing chemicals (e.g. e-cigarettes) induce inflammation, oxidative stress, alteration of mucociliary clearance and DNA injury [11]. Moreover, previous human and animal studies have reported that exposure to flavoured WPS causes pulmonary inflammation, oxidative stress and alterations in lung function and morphology [12,13,14,15,16]. With respect to the cardiovascular effects of WPS, clinical studies have demonstrated that short-term WPS affects the heart rate, blood pressure, tissue oxygenation and vascular function and that the long-term use of WPS increases the risk of coronary artery disease [6,17]. We have reported experimental evidence in mice that WPS inhalation causes cardiovascular oxidative stress, inflammation and thrombotic disorders in mice [13,18]. However, the effect of flavouring on the cardiovascular effects of WPS inhalation remains unclear. Therefore, the present study was designed to assess the possible effect of flavouring on the cardiovascular pathophysiological impact of WPS and the mechanisms behind these actions. 

## 2. Results

### 2.1. Systolic Blood Pressure (SBP) 

**Figure 1:**
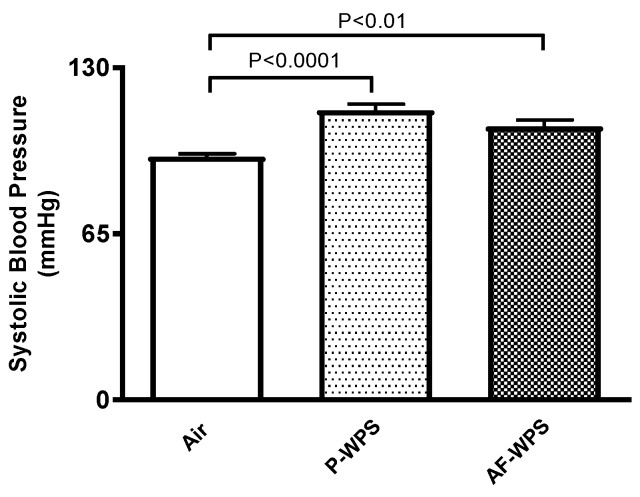
Effects of plain water-pipe smoke (P-WPS) or apple-flavoured (AF)-WPS on systolic blood pressure. Systolic blood pressure measured in mice at the end of 1 mo exposure period to either air or P-WPS or AF-WPS. Data are mean ± SEM (n = 6); one-way ANOVA followed by Holm–Sidak’s multiple comparison test.

Figure 1 illustrates the results of non-invasive measurement of SBP in mice. The data shows that compared with the control group, mice exposed to either unflavoured, i.e. plain (P)-WPS (P < 0.0001) or apple-flavoured (AF)-WPS (P < 0.01) showed a significant increase in SBP.

### 2.2. Thrombotic Occlusion Time in Pial Microvessels in Vivo 

**Figure 2:**
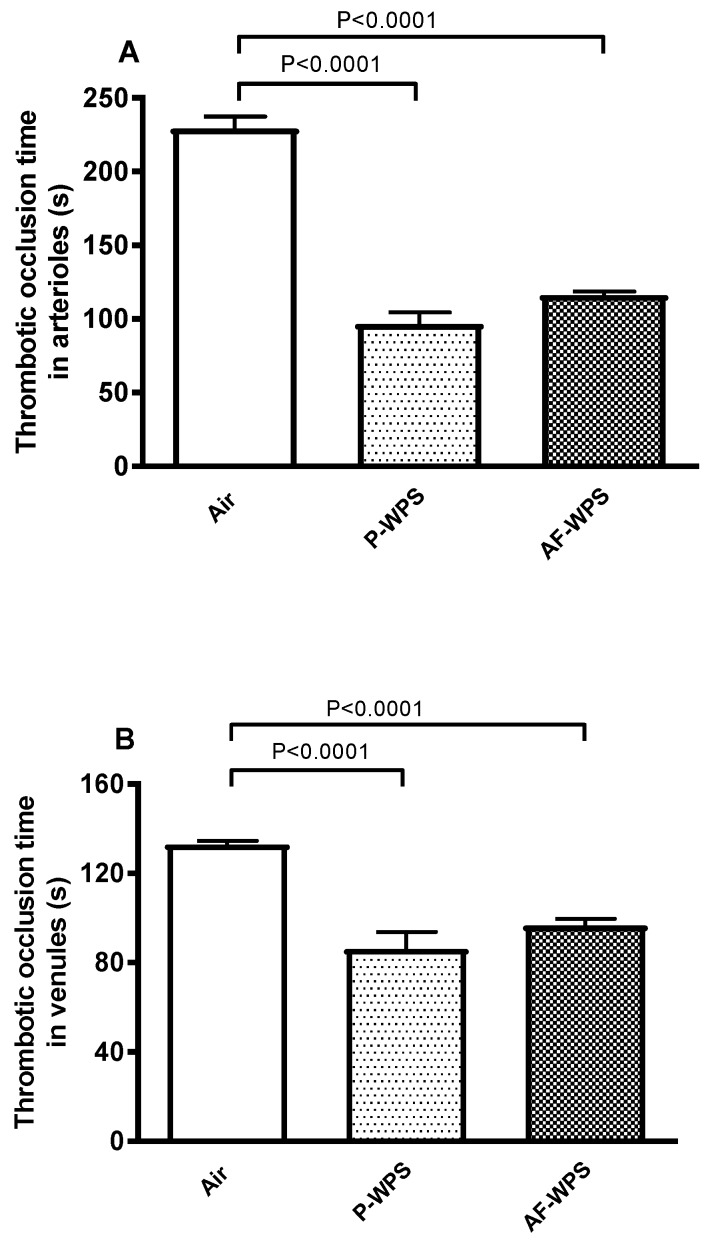
Effects of plain water-pipe smoke (P-WPS) or apple-flavoured (AF)-WPS on thrombosis in vivo. Thrombotic occlusion time in pial arterioles (**A**) and venules (**B**) in mice at the end of 1 mo exposure period to either air or P-WPS or AF-WPS. Data are mean ± SEM (n = 6); one-way ANOVA followed by Holm–Sidak’s multiple comparison test.

Figure 2 shows the in vivo measurement of thrombotic occlusion time in pial arterioles and venules of mice. Inhalation of either P-WPS or AF-WPS induced prothrombotic effects in both pial arteriols and venules (Figure 2). Compared with air-exposed mice, the thrombotic occlusion time in pial arterioles was shortened by both P-WPS (P < 0.0001) and AF-WPS (P < 0.0001) (Figure 2A). Likewise, in venules, exposure to either P-WPS (P < 0.0001) or AF-WPS (P < 0.0001) induced a significant shortening of the thrombotic occlusion time (Figure 2B).

### 2.3. Platelet Aggregation in Vitro 

**Figure 3:**
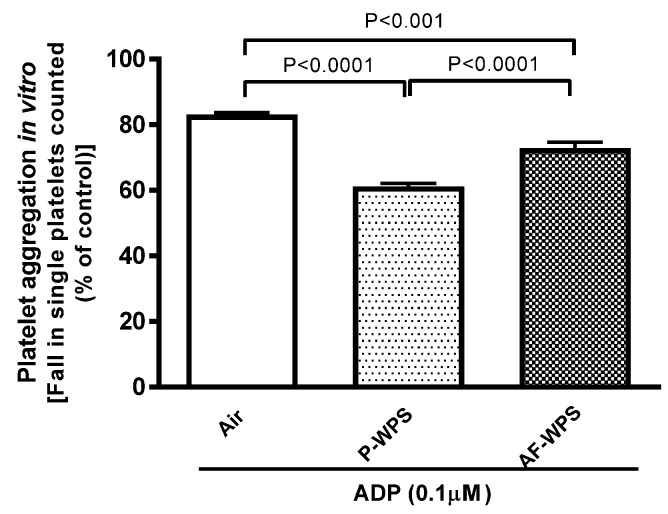
Effects of plain water-pipe smoke (P-WPS) or apple-flavoured (AF)-WPS on platelet aggregation in vitro. In vitro platelet aggregation in whole blood obtained after the addition of adenosine diphosphate (ADP, 0.1 μM ) to whole blood collected from mice at the end of 1 mo exposure period to either air or P-WPS or AF-WPS. Data are mean ± SEM (n = 5); one-way ANOVA followed by Holm–Sidak’s multiple comparison test.

Figure 3 illustrates the evaluation of platelet aggregation in whole blood in vitro. Following the incubation with adenosine diphosphate (ADP), there was a significant fall in single platelets, indicating the occurrence of platelet aggregation in whole blood of mice exposed to either P-WPS (P < 0.0001) or AF-WPS (P < 0.001) compared with the group of mice exposed to air only. Moreover, there was a statistical difference between P-WPS and AF-WPS groups (P < 0.0001). 

### 2.4. Prothrombin Time (PT) and Activated Partial Thromboplastin Time (aPTT)

**Figure 4:**
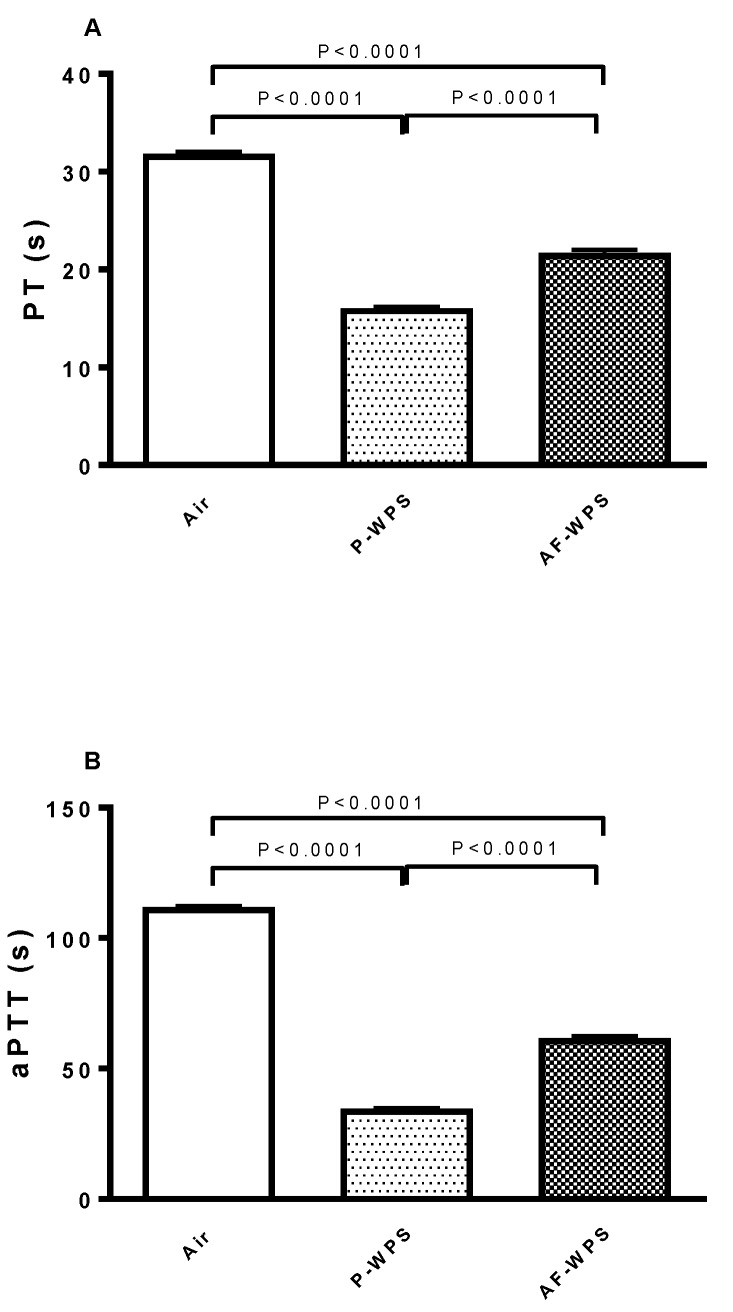
Effects of plain water-pipe smoke (P-WPS) or apple-flavoured (AF)-WPS on prothrombin time (PT, A) and activated partial thromboplastin time (aPTT) in vitro. PT (**A**) and aPTT (**B**) at the end of 1 mo exposure period to either air or P-WPS or AF-WPS. Data are mean ± SEM (n = 6); one-way ANOVA followed by Holm–Sidak’s multiple comparison test.

Figure 4 displays the assessment of PT which evaluate the production of the fibrin clot via the activity of the extrinsic and common coagulation pathways and aPTT which estimates the activity of the intrinsic and common pathways of coagulation. Compared with the air-exposed group, inhalation of either P-WPS or AF-WPS for 1 mo induced a significant shortening of PT (P < 0.0001) and aPTT (P < 0.0001). Moreover, there was a statistical difference between P-WPS and AF-WPS groups (P < 0.0001). 

### 2.5. Fibrinogen and von Willebrand Factor (vWF) Concentrations in Plasma

**Figure 5:**
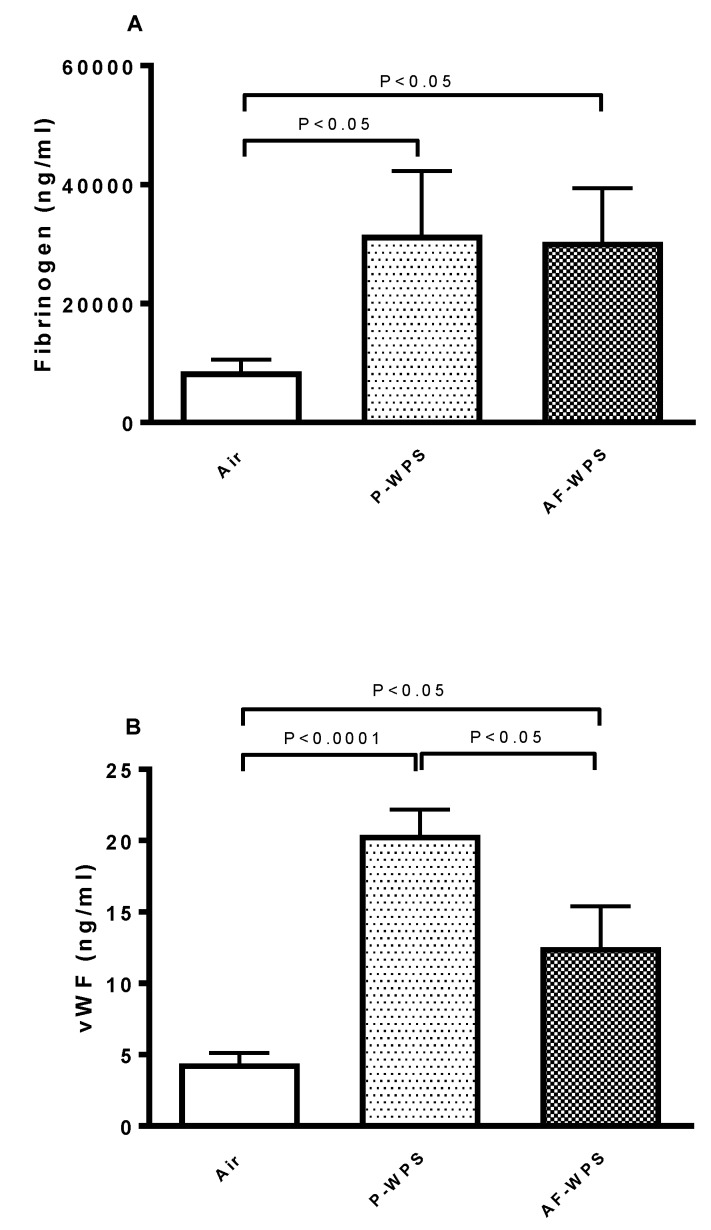
Effects of plain water-pipe smoke (P-WPS) or apple-flavoured (AF)-WPS on plasma concentration of fibrinogen and von Willebrand factor (vWF). Plasma concentrations of fibrinogen (**A**) and von vWF (**B**) at the end of 1 mo exposure period to either air or P-WPS or AF-WPS. Data are mean ± SEM (n = 7–8). Fibrinogen was analysed with Kruskal–Wallis test followed by Dunn’s multiple comparison and vWF was analysed by one-way ANOVA followed by Holm–Sidak’s multiple comparison test.

Figure 5 shows the concentration of fibrinogen, which is known to promote thrombosis, and vWF, which mirrors blood vessel reactivity. The exposure to either P-WPS or AF-WPS caused a significant increase in the concentrations of fibrinogen and vWF. Moreover, for vWF, there was a statistical difference between P-WPS and AF-WPS groups. 

### 2.6. Markers of Oxidative Stress in Heart Homogenates 

**Figure 6:**
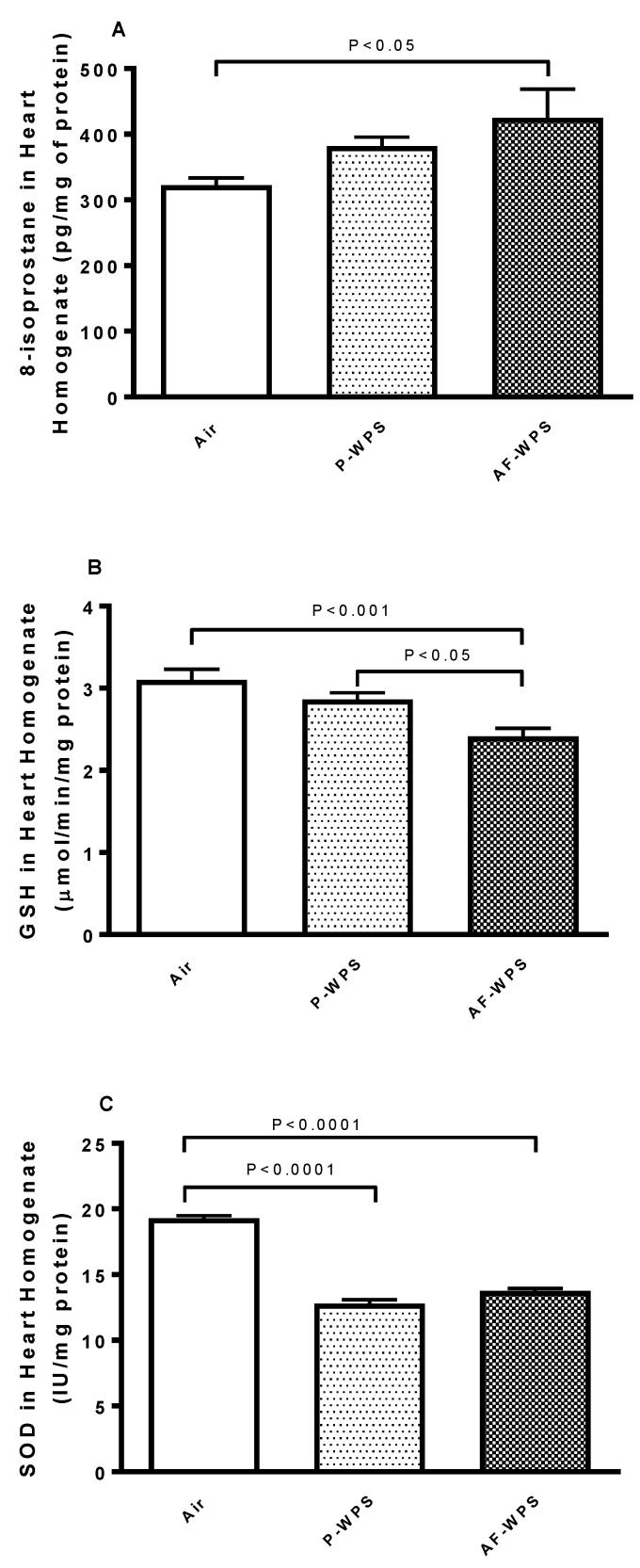
Effects of plain water-pipe smoke (P-WPS) or apple-flavoured (AF)-WPS on markers of oxidative stress. Heart homogenate levels of 8-isoprostane (**A**), reduced glutathione (GSH, **B)** and superoxide dismutase (SOD, **C**) at the end of 1 mo exposure period to either air or P-WPS or AF-WPS. Data are mean ± SEM (n = 6–8); one-way ANOVA followed by Holm–Sidak’s multiple comparison test.

Figure 6 exemplifies the heart homogenate levels of 8-isoprostane, a marker of lipid peroxidation, and those of the antioxidants reduced glutathione (GSH) and superoxide dismutase (SOD). While the inhalation of P-WPS for 1 mo failed to increase significantly the concentration of the marker of lipid peroxidation, 8-isoprostane, exposure to AF-WPS induced a significant augmentation of 8-isoprostane concentration in heart homogenates (P < 0.05) (Figure 6A). Likewise, compared with the control group, the decrease of the concentration of antioxidant GSH reached a statistical significance only in the group of mice exposed to AF-WPS (P < 0.001) (Figure 6B). Moreover, there was a statistical difference between P-WPS and AF-WPS groups (P < 0.05). Regarding SOD, compared with air-exposed mice, the activity of SOD was significantly decreased in both P-WPS (P < 0.0001) and AF-WPS (P < 0.0001) groups. 

### 2.7. Markers of Inflammation in Heart Homogenates 

**Figure 7:**
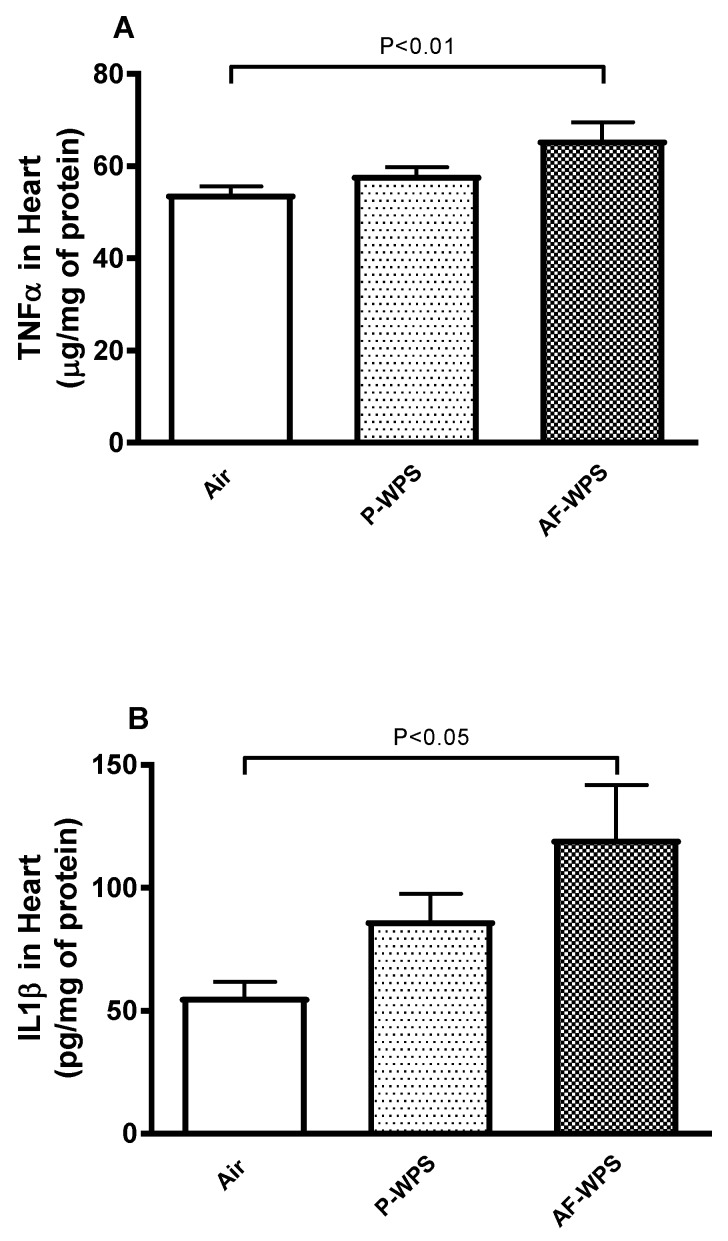
Effects of plain water-pipe smoke (P-WPS) or apple-flavoured (AF)-WPS on markers of inflammation. Heart homogenate concentrations of tumour necrosis factor α (TNFα, **A**) and interleukin 1β (IL-1β, **B**) at the end of 1 mo exposure period to either air or P-WPS or AF-WPS. Data are mean ± SEM (n = 6–8); one-way ANOVA followed by Holm–Sidak’s multiple comparison test.

The assessment of the proinflammatory cytokines tumour necrosis factor α (TNFα) and IL-1β is shown in Figure 7. The latter illustrates that, compared with the control group, inhalation of AF-WPS for 1 mo induced an increase in the concentrations of TNFα (P < 0.01) and interleukin 1β (IL-1β) (P < 0.05) in heart homogenates. However, inhalation of P-WPS failed to augment significantly the concentration of TNFα and IL-1β.

### 2.8. Troponin I Concentration, DNA Damage and Cleaved Caspase-3

**Figure 8:**
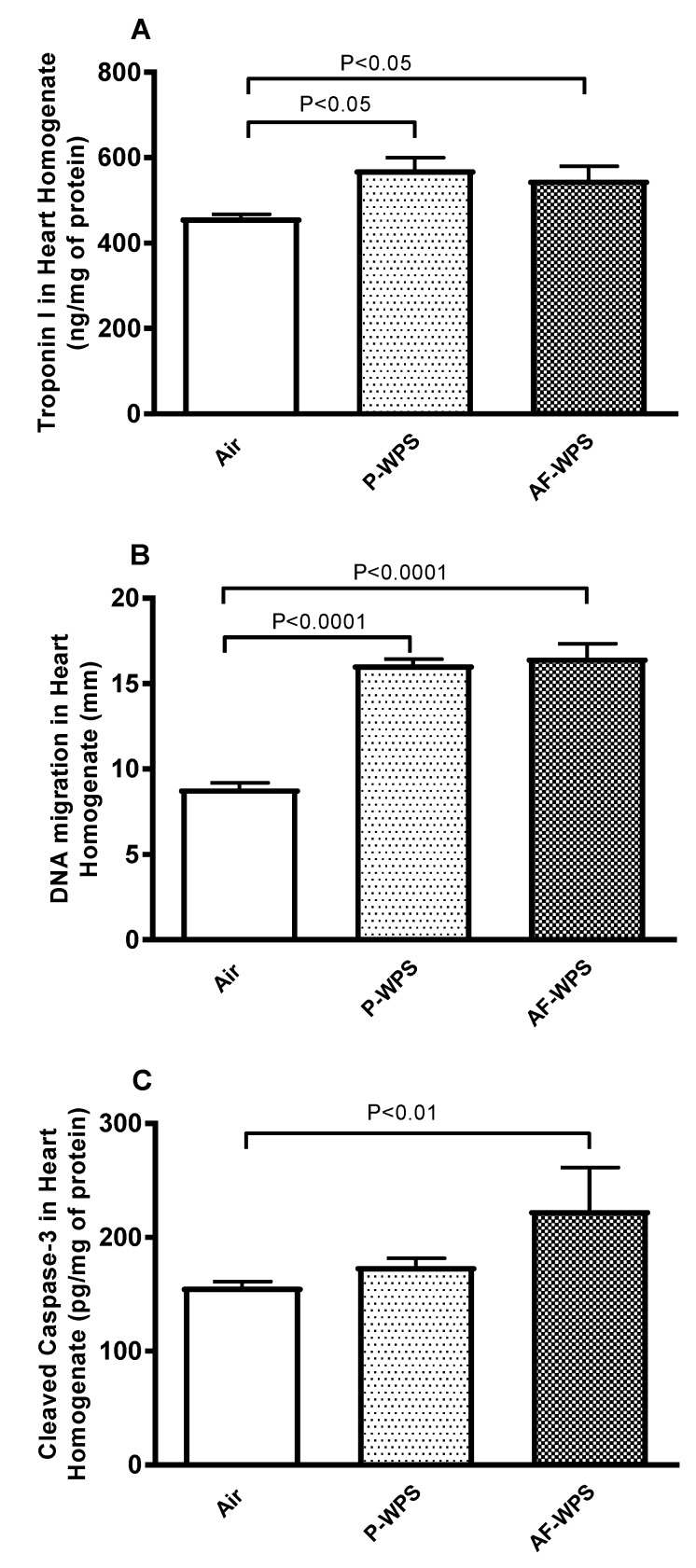
Effects of plain water-pipe smoke (P-WPS) or apple-flavoured (AF)-WPS on heart injury, DNA damage and apoptosis. Heart homogenate concentrations of troponin I (**A**), DNA migration (mm) in the heart assessed by Comet assay (**B**) and cleaved caspase-3 concentration (**C**) in heart homogenates at the end of 1 mo exposure period to either air or P-WPS or AF-WPS. Data are mean ± SEM (n = 5–7). Troponin I and DNA migration were analysed by one-way ANOVA followed by Holm–Sidak’s multiple comparison test, and cleaved caspase-3 was analysed with Kruskal–Wallis test followed by Dunn’s multiple comparison test.

Figure 8 displays the assessment of markers of myocardial injury (Troponin I), DNA damage and apopotosis (cleaved caspase-3). Figure 8A,B shows that exposure to either P-WPS or AF-WPS caused an increase in the concentration of troponin I (P < 0.05) in heart homogenates and induced DNA damage (P < 0.0001) measured by COMET assay. Figure 8C shows that the exposure to AF-WPS caused a significant increase in cleaved caspase-3 (P < 0.01). However, the increase of cleaved caspase-3 concentration induced by P-WPS inhalation was slight and failed to reach statistical significance (Figure 8C).

### 2.9. Heart Histology and Immunohistochemistry

**Figure 9:**
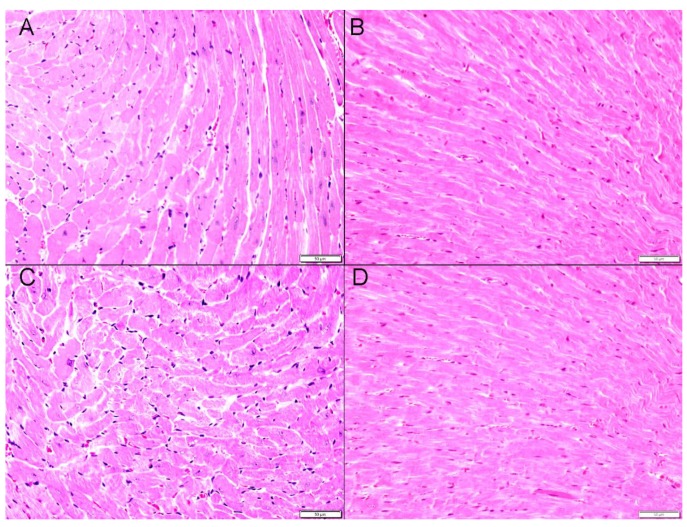
Effects of plain water-pipe smoke (P-WPS) or apple-flavoured (AF)-WPS on heart morphology. Representative light microscopy sections of hematoxylin and eosin stained heart tissues of mice at the end of 1 mo exposure period to either air (**A**,**B**) or P-WPS (**C**) or AF-WPS (**D**). (**A**,**B**) Representative heart sections obtained from air-exposed mice showing unremarkable heart morphology and architecture. (**C**) Representative heart sections obtained from P-WPS-exposed mice showing unremarkable heart morphology and architecture. (**D**) Representative heart sections obtained from AF-WPS-exposed mice showing unremarkable heart morphology and architecture. The scale bar equals 50 μm.

**Figure 10:**
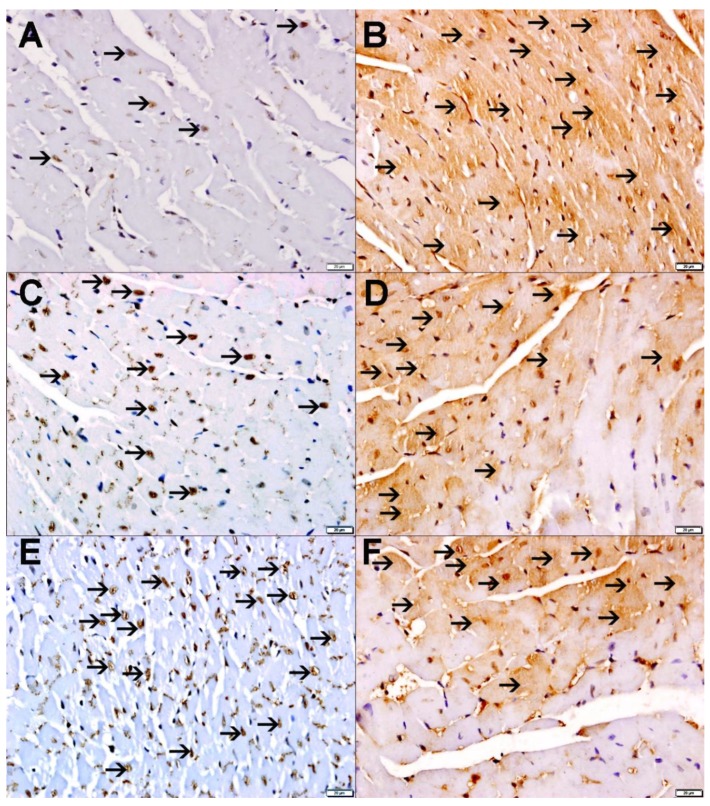
Effects of plain water-pipe smoke (P-WPS) or apple-flavoured (AF)-WPS on the immunohistochemistry detection of nuclear factor erythroid-derived 2-like 2 (Nrf2) or superoxide dismutase (SOD) in the heart. Immunohistochemical analysis of the heart tissue sections of mice for the detection of Nrf2 (**A**,**C**,**E**) and SOD (**B**,**D**,**F**) at the end of 1 mo exposure period to either air or P-WPS or AF-WPS. (**A**) Representative section of the heart of air-exposed mice showing focal mild nuclear expression of Nrf2 by cardiac myocytes (arrow). (**B**) Representative section of the heart of air-exposed mice showing diffuse cytoplasmic expression of SOD by cardiac myocytes (arrow). (**C**) Representative section of the heart of P-WPS-exposed mice showing nuclear expression of Nrf2 by cardiac myocytes (arrow). (**D**) Representative section of the heart of mice exposed to P-WPS showing cytoplasmic expression of SOD by cardiac myocytes (arrow). (**E**) Representative section of the heart of AF-WPS-exposed mice showing nuclear expression of Nrf2 by cardiac myocytes (arrow). (**F**) Representative section of the heart of mice exposed to AF-WPS showing cytoplasmic expression of SOD by cardiac myocytes (arrow). The scale bar equals 50 μm.

The evaluation of heart morphology and the quantification by immunohistochemistry of the oxidant-stress sensing transcription factor nuclear factor erythroid-derived 2-like 2 (Nrf2) and the antioxidant SOD are shown in Figure 9 and Figure 10. There were no morphological changes in hematoxylin and eosin (H and E) stained sections of hearts obtained from mice exposed to air or P-WPS or AF-WPS (Figure 9).

Figure 10A,C,E reveals that there is nuclear and cytoplasmic expression of Nrf2 by cardiac myocytes in the heart sections of the studied groups with various intensity and distribution. Morphometrical analysis of the nuclear expression of Nrf2 revealed that air-exposed group showed mild nuclear expression of Nrf2 by a few cardiomyocytes (15.6 ± 1.59%) and scored 1 (Figure 10A). When compared with the control group, the P-WPS-exposed group showed an increase in the nuclear expression of Nrf2 by cardiomyocytes (46.5 ± 2.1%, P < 0.0001) and scored 2 (Figure 10C). Mice exposed to AF-WPS revealed higher nuclear expression of Nrf2 by cardiomyocyte (59.2 ± 2.3) when compared with either the P-WPS (*P* < 0.01) or control (P < 0.0001) groups and scored 3 (Figure 10E).

Figure 10B shows that there was almost a diffuse cytoplasmic expression of SOD by cardiomyocytes in the air-exposed group (80.5 ± 6.0%) and scored 4. In P-WPS-exposed mice, there was a lower expression of SOD by cardiomyocytes (65.1 ± 2.4, P < 0.05) when compared with the air-exposed group and scored 3 (Figure 10D). In the hearts of mice exposed to AF-WPS, there was a lower expression of SOD by cardiomyocyte (55.6 ± 2.4, P < 0.01) when compared with the control group and scored 3 (Figure 10F).

### 2.10. Quantification of Heme Oxygenase-1 (HO-1)

**Figure 11:**
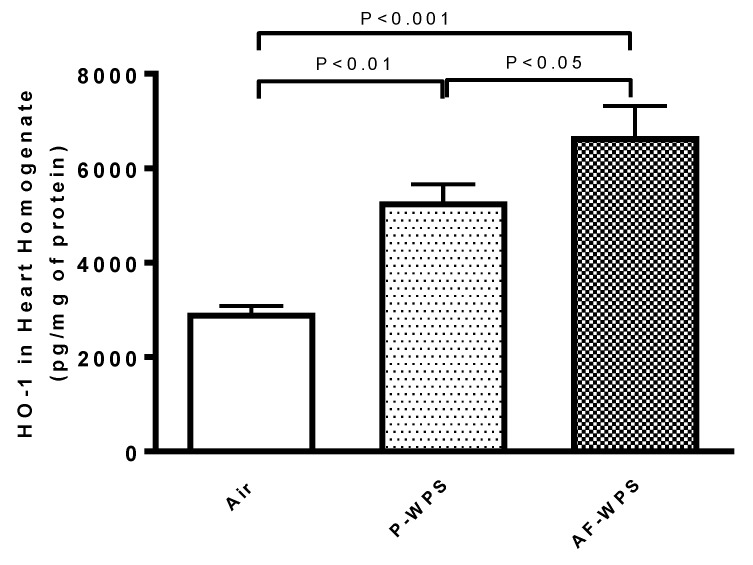
Effects of plain water-pipe smoke (P-WPS) or apple-flavoured (AF)-WPS on heme oxygenase-1 (HO-1) concentrations in heart. Heart homogenate concentrations of HO-1 at the end of 1 mo exposure period to either air or P-WPS or AF-WPS. Data are mean ± SEM (n = 5); one-way ANOVA followed by Holm–Sidak’s multiple comparison test.

The estimation of HO-1, an Nrf2-regulated gene, which exerts an important role in the mitigation of oxidative stress and inflammation, is illustrated in Figure 11. The concentration of HO-1 was significantly increased in the heart homogenates of both P-WPS and AF-WPS groups compared with air-exposed group. Moreover, the effect seen in AF-group was significantly higher than that observed in P-WPS group.

### 2.11. Measurement of Cotinine

**Figure 12:**
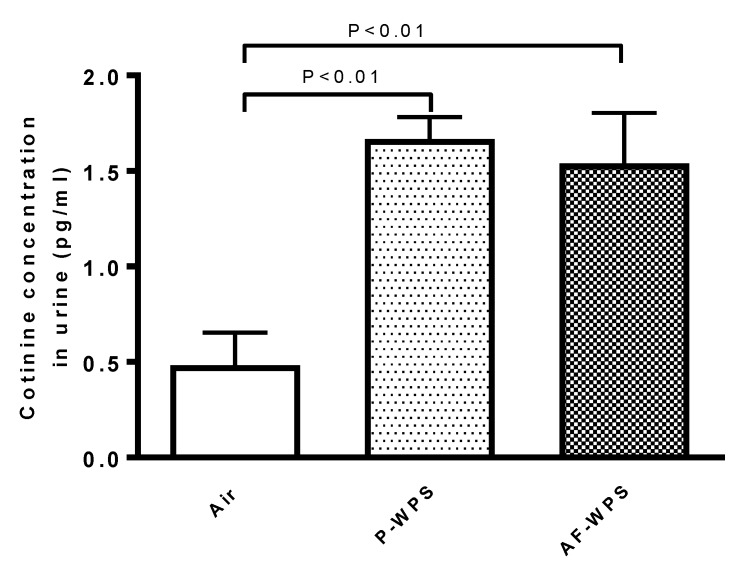
Effects of plain water-pipe smoke (P-WPS) or apple-flavoured (AF)-WPS on cotinine concentrations in urine. Cotinine concentrations measured in urine at the end of 1 mo exposure period to either air or P-WPS or AF-WPS. Data are mean ± SEM (n = 5); one-way ANOVA followed by Holm–Sidak’s multiple comparison test.

The quantification of cotinine, the main metabolite of nicotine is shown in Figure 12. Compared with air-exposed group, cotinine concentration in urine was significantly increased in both P-WPS (P < 0.01) and AF-WPS (P < 0.01).

## 3. Discussion

We showed that inhalation of both P-WPS and AF-WPS induced a hypercoagulable state, increased systolic blood pressure, troponin I, oxidative stress and DNA damage in heart homogenates. Of note, the expression of Nrf2 and oxidative stress markers in the heart were more consistently affected by AF-WPS inhalation. Moreover, markers of inflammation (TNFα and IL1β) and cleaved caspase-3 were only significantly increased by AF-WPS exposure.

The acceptance of WPS has considerably increased since the introduction of various flavouring in tobacco such as fruit (for example, apple, strawberry, grape and melon), menthol/mint, sweet substances (candy, chocolate) and spices (for example, clove) [6,19]. The attractiveness of flavoured tobacco has been reported to be the main reason why young people begin smoking water-pipe and the misperception that flavoured tobacco products are less toxic [19]. In fact, flavoured tobacco is frequently used by waterpipe consumers, and in the USA, it has been reported that 82.3% of adults who smoked waterpipe in the past 30 days stated using a flavoured tobacco [20]. The same study has shown that the flavouring use was analogous between women (83.6%) and men (81.3%) [20]. Nevertheless, studies on the cardiovascular toxicity of the flavourings used in WPS are very scarce and are much needed. Experimental studies on WPS are essential because they give biological credibility to the impact of WPS in humans. Moreover, they allow the study of the exclusive effects of WPS. These have been shown to be problematic in humans because most waterpipe users are either current or former cigarette smokers [21]. 

It has been recently demonstrated that the exposure of mice to WPS for either 1 or 6 mo causes a significant increase in the SBP [13,18]. However, a comparison between the impact of unflavoured and AF-WPS on SBP has not been investigated so far. Here, we show that exposure of mice to either P- or AF-WPS induces a significant increase in SBP. Several clinical studies have reported that WPS induces an increase in systolic blood pressure. This effect has been related to the sympathomimetic effect of nicotine [22,23].

Similarly to the SBP, our data reveal that inhalation of either unflavoured or AF-WPS induces a prothrombotic state, substantiated in vivo and in vitro. We also found an increase in the plasma concentrations of fibrinogen and vWF. Fibrinogen is an acute-phase protein which enhances blood viscosity and promotes thrombosis, and vWF indicates vascular reactivity and plays a key role in platelet adhesion to injured endothelium [24]. The latter possibly explains the prothrombotic effects of P- and AF-WPS observed in vivo in pial arterioles and venules. While both P-WPS and AF-WPS were effective in inducing procoagulant effects, we found that for some measured parameters (platelet aggregation in vitro, PT, aPTT and vWF), the effects observed in P-WPS were statistically higher than those seen in AF-WPS group. It is well-established that exposure to CS triggers platelet activation, stimulates the coagulation cascade, and increases in the concentrations of fibrinogen and vWF [24,25]. Moreover, a recent human study reported that both WPS and CS caused significant increases in the plasma levels of fibrinogen, factor VII and factor VIII, and the degree of increase of fibrinogen observed in waterpipe smokers was higher than that observed in cigarette smokers [26]. Nevertheless, as far as we are aware, no human study has compared the impact of flavoured versus unflavoured WPS with respect to thrombosis. 

Oxidative stress reflects a disruption in the balance between the generation of reactive oxygen species (free radicals) and antioxidant defences, with the consequence being favourable to oxidants [27]. Both clinical and experimental studies have reported the occurrence of oxidative stress following acute and chronic exposure to WPS [13,14,18,28,29]. In the present study, we show that only AF-WPS inhalation caused a significant increase of 8-isoprostane, a marker of lipid peroxidation, and a decrease of reduced glutathione. However, both unflavoured and AF-WPS exposure decreased the activity of the antioxidant SOD. The decreases of antioxidants suggest the depletion of antioxidant protections [30,31]. Oxidative stress and inflammation are narrowly interrelated in pathophysiological processes affecting the cardiovascular system [32]. In line with our oxidative stress findings, we found no statistically significant increase in TNFα and IL-1β in mice exposed to unflavoured WPS. However, inhalation of AF-WPS induced a substantial increase in the pro-inflammatory cytokines TNFα and IL-1β in heart homogenates. It has been recently reported that flavouring chemicals present in e-cigarette aerosols are able to trigger the release of reactive oxygen species and induce inflammation in human lung epithelial cells and fibroblasts [27]. Moreover, it has been shown that flavouring contributes substantially to the alteration in lung cell viability and the respiratory barrier integrity induced by e-cigarettes, in particular the flavouring based on natural plant extracts such as Sambuca [33]. Moreover, using cultured myocardial cells, it has been shown that the cytotoxicity of e-cigarettes is related to the use of flavouring [34]. 

Our data demonstrate that the concentration in heart homogenates of troponin I, a biomarker of myocardial damage, was significantly elevated in mice exposed to either AF-WPS or unflavoured WPS inhalation, indicating the occurrence of myocardial damage and cardiotoxicity. Likewise, the DNA damage evaluated by COMET assay was shown to be increased following exposure to both unflavoured WPS and AF-WPS. The latter effects can be explained by the occurrence of oxidative stress observed following inhalation of unflavoured (decrease of SOD) and AF-WPS (increase of 8-isoprostane and decrease of both GSH and SOD). Both animal and clinical studies have reported DNA damage and oxidative stress in mice exposed to WPS and healthy subjects who smoke waterpipe [13,28,35]. 

It is well-known that cardiac apoptosis is increased in cardiotoxicity, heart failure and physiological aging and is considered as a marker of poor cardiovascular outcomes [36,37]. Moreover, apoptosis is related to augmented DNA damage and/or impaired DNA repair systems [36,37]. It has been shown that oxidative stress is capable of regulating apoptosis typically through caspase-3 activation [38]. Our data show that while inhalation of AF-WPS increased the concentration of caspase-3, the exposure to unflavoured WPS did not elevate caspase-3 concentration. The latter finding could be explained by the fact that AF-WPS exposure has triggered oxidative stress more potently by increasing 8-isoprostane and decreasing GSH and SOD, whereas unflavoured WPS decreased SOD. Additional studies are needed to clarify this point.

In line with the results obtained in heart homogenates, the immunohistochemical analysis of the hearts revealed that both unflavoured WPS and AF-WPS inhalation decreased the expression of SOD by cardiomyocytes. This effect suggests that this antioxidant is being depleted by the exposure of WPS. Likewise, following exposure to either AF-WPS or unflavoured, the expression of Nrf2 at nuclear level by cardiomyocytes was higher compared with air-exposed mice. Nrf2 is an oxidant-stress sensing transcription factor which is ordinarily stored in the cytoplasm via interaction with kelch-like ECH-associated protein 1. However, this association is interrupted following electrophilic attack, permitting Nrf2 to move into the nucleus and initiate gene expression [39]. Therefore, Nrf2 activation develops following the occurrence of oxidative stress and leads to the activation of anti-oxidant enzymes that contribute to protection against toxicants [40]. Furthermore, our data show that the nuclear expression of Nrf2 in the heart of mice that inhaled AF-WPS was higher than those that inhaled unflavoured WPS. Increases of Nrf2 expression have been reported following exposure to WPS and CS and have been used as an essential therapeutic target in various cardiovascular and respiratory diseases for their induction of anti-oxidant enzymes and other protective enzymes [35,41,42]. In parallel with Nrf2, we observed a significant increase in the concentration of HO-1 in both P-WPS and AF-WPS groups compared with control group, and the effect seen in AF-group was higher than that observed in P-WPS group. HO-1 is known to play an important antioxidant role in the cardiovascular system [43]. The intensification of both nuclear Nrf2 expression and the increase of HO-1 in the heart seen in mice exposed to AF-WPS could be explained by possible compensatory mechanism aimed at reducing the high degree of oxidative stress caused by the exposure to AF-WPS.

It has been reported that WPS in humans induces a five-fold increase in plasma nicotine concentration, i.e., from 3 ± 3 ng/ml earlier to smoking to 15 ± 8 ng/ml following a water-pipe smoking session [44]. Cotinine, which has been measured in the current study, is a metabolite of nicotine that is usually utilized as biochemical marker of smoking status in studies related to tobacco cessation [45]. The latter biomarker was reported to increase in the urine of water-pipe smokers [46]. While the concentration of cotinine did not differ between the group of mice exposed for 1 mo (subchronic exposure) to unflavoured (1.66 ± 0.11 pg/ml) versus AF-WPS (1.54 ± 0.26 pg/ml), it was significantly increased in P-WPS (3.5-fold increase) and AF-WPS (3.2-fold increase) compared with air-exposed mice (0.48 ± 0.17 pg/ml). We have recently reported a higher increase of cotinine (19-fold increase) in mice chronically (6 mo) exposed to WPS (8.3 ± 2.6 pg/ml) compared with control group (0.40 ± 0.06 pg/ml) [47]. In humans smoking water-pipe, the concentration of cotinine in urine is much higher (25-fold increase) compared with non-smokers (median 162.7 versus 6.5 ng/ml) [48]. It remains to establish the effects of unflavoured WPS versus AF-WPS following chronic exposure to WPS. 

The toxicity of flavours could depend on chemical ingredients and the presence of harmful and potentially harmful constituents in tobacco products [11]. A study has reported the existence of 79 volatile flavouring compounds in water-pipe tobacco including large quantities of benzyl alcohol along with high levels of linalool, eugenol and limonene [49,50]. Moreover, the chemical analysis of mainstream smoke of WPS by Perraud et al. [51] revealed the presence of CO which is generated principally from the charcoal utilized to heat the tobacco and volatile organic compounds, consisting mainly in glycerol and its breakdown products, for instance, benzene, acetaldehyde and acrolein. Moreover, significant concentrations of ultrafine particles (4–100 nm) constituted of sugar derivatives were also found during WPS session [51]. Additional studies are required to evaluate the chemical composition and nanoparticles emission following exposure to WPS.

## 4. Material and Methods

### 4.1. Animals Experimentation 

All animal experimentation protocols were approved (21 June 2017) by the Institutional Animal Care and Use Committee of United Arab Emirates University [Approval # ERA_20175625].

### 4.2. WPS Exposure

BALB/C mice of both genders, aged 6–8 weeks, weighing 20–25 g (Taconic Farms Inc., Germantown, NY, USA) were bred in the local central animal facility of the College of Medicine and Health Sciences, United Arab Emirates University. The animals were maintained in a temperature-controlled facility, with a 12-h light/dark cycle, and were provided access to water and food ad libitum.

After one week of familiarization to their conditions, mice were indiscriminately separated into 3 groups, air (control), P-WPS and AF-WPS. Depending on the type of experiment performed, the number of animals used in each group ranged between n = 5 and n = 8. The number of mice used in each measured parameter is indicated in the figure legends. Mice were placed in soft restraints and attached to the exposure tower [13,18]. The WPS inhalation protocol has been performed according to the methods that we have recently described [13,18]. Mice were exposed using nose-only exposure system (InExpose System, Scireq, Montreal, QC, Canada) to mainstream WPS generated by commercially available P tobacco or AF tobacco (Al Fakher Tobacco Trading, Ajman, UAE). The tobacco was ignited using instant light shisha charcoal (Al Qaed International General Trading, Dubai, UAE). Similarly to its use in humans, the aspiration effect exercised by the pump causes the smoke from the waterpipe to initially pass through the water before it reaches into the exposure tower [13,18]. Control mice were exposed to air only. The duration of the session was 30 min/day and 5 days/wk for 1 mo. The inhalation procedure was monitored by a computerized system. A computer-monitored puff was produced every 1 min (consisting of a 2 s puff time of WPS after that a 58 s of fresh air) (InExpose System, Scireq, Montreal, QC, Canada). A detailed description and a schematic representation of the exposure system has been recently reported [13]. 

### 4.3. SBP Measurement

SBP was assessed in the air, P-WPS and AF-WPS groups using a noninvasive computerized tail-cuff system for measuring blood pressure in mice (ADInstrument, Colorado Springs, CO, USA), according to recently described technique [13,18]. 

### 4.4. Experimental Pial Cerebral Arterioles Thrombosis Model

In vivo pial arteriolar and venular thrombogenesis was assessed at the end of the 1 mo exposure period to either P-WPS or AF-WPS or air, according to a previously described technique [13,18]. 

### 4.5. Platelet Aggregation in Mouse Whole Blood.

In separate mice exposed to either P-WPS or AF-WPS or air, the platelet aggregation assay in whole blood was performed as described before [13,18]. 

### 4.6. PT and aPTT Measurement in Plasma in Vitro

In separate animals, at the end of the one-month exposure period to P-WPS or AF-WPS or air, blood was withdrawn from mice, and PT and aPTT were measured according to a previously described technique [13,18].

### 4.7. Measurement of Fibrinogen and vWF Concentrations in Plasma

At the end of the exposure period, separate mice were anaesthetised with an i.p. injection of sodium pentobarbital (45 mg/kg), and then, blood was collected from the inferior vena cava in EDTA (4 %), and after that it was centrifuged for 15 min at 4 ºC at 900 *g*, and the plasma samples obtained were frozen at –80 °C pending analysis. Fibrinogen and vWF concentrations in plasma were estimated according to the manufacturer’s instructions supplied in the commercially available assay kits acquired from Molecular Innovation (Southfield, MI, USA) and Cloud-Clone Corp. (Houston, TX, USA).

### 4.8. Measurement of 8-isoprostane, GSH, SOD, TNFα and IL1β Levels in Heart Homogenates

The preparation of heart homogenates for the quantification of inflammatory and oxidative stress markers was performed as described before [13,18]. Protein content in each sample was measured by Bradford’s method, as described earlier [13,18]. The concentrations of 8-isoprostane and the SOD activity were quantified according to the manufacturer’s instructions provided in the commercially available assay kits obtained from Cayman Chemicals (Michigan, USA). The levels of the pro-inflammatory cytokines TNFα and IL1β were measured by enzyme-linked immunosorbent assays using commercially available kits obtained from R&D systems (Duo Set, Minneapolis, MN, USA. Troponin I concentration was determined according to the protocol supplied in the commercially available assay kit acquired from Life Diagnostics (West Chester, PA, USA).

### 4.9. Assessment of DNA Damage in the Heart by COMET Assay 

In separate mice, the hearts collected at the end of the one-month exposure duration to either air or P-WPS or AF-WPS were used to quantify the DNA damage by COMET assay. The latter was performed as reported earlier [13]. 

### 4.10. Measurement of Cleaved Caspase 3 in Heart Homogenates

The quantification of cleaved caspase 3 in heart homogenates obtained from mice exposed to either air or P-WPS or AF-WPS were prepared as previously reported [13,18]. Cleaved caspase 3 was measured in air, P-WPS and AF-WPS groups with mouse (Asp175) DuoSet IC ELISA kit purchased from R&D systems (Minneapolis, MN, USA).

### 4.11. Histopathology and Immunohistochemistry

After fixation of heart tissue in neutral buffered formalin (10% *w*/*v*) for a week, the tissue was gradually dehydrated in increasing concentrations of ethanol, cleared of alcohol residue in xylene, and finally embedded in paraffin. Sections of 3 μm were cut using a microtome (RM2125 RTS, Leica Biosystems, Nussloch, Germany) and stained with H and E. Sections were mounted on slides and evaluated blindly using light microscopy by a histopathologist who participated in this project (SA) using a 40× objective lens.

The immunohistochemical preparation and analysis of heart section for the detection of nuclear factor erythroid 2–related factor 2 (NRF-2) and SOD were performed using anti-NRF-2 (Rabbit Polyclonal, 1:100, Abcam, Burlingame, CA, USA) and anti-SOD (Rabbit Polyclonal, 1:300, Abcam, Burlingame, CA, USA) antibodies, as described before [35,52]. The immunohistochemical staining of the heart tissue was scored semi-quantitatively on a scale of 0–4 according to % of staining of heart muscles in 4 slides of each specimen, and each slide included 4 equal coronal slices of the heart. A score of 0 was given if the expression varied from 0% to 10%, 1 for 11% to 25%, 2 for 26% to 50%, 3 for 51% to 75% and 4 for over 75% [35,52].

### 4.12. Measurement of HO-1 in Heart Homogenates

The measurement of heme oxygenase-1 in heart homogenates obtained from mice exposed to air or P-WPS or AF-WPS was performed using an ELISA kit procured from Abcam (Burlingame, CA, USA).

### 4.13. Cotinine Measurement

The quantification of urinary cotinine concentrations was performed using an ELISA kit purchased from Creative Diagnostics (Shirley, NY, USA). The latter was assessed following the final exposure session to P-WPS or AF-WPS or air on urine samples obtained overnight by means of metabolic cages. 

### 4.14. Statistics 

GraphPad Prism Version 7.03 for Windows software (Graphpad Software Inc., San Diego, USA) was used to perform the statistical analysis and generate the graphs. Normality of distribution was assessed by the Shapiro–Wilk statistic normality test. Comparisons between groups were performed by one-way analysis of variance (ANOVA), followed by Holm–Sidak’s multiple comparison post hoc test. Data which were not normally distributed (concentrations of fibrinogen in plasma and cleaved caspase-3 in heart homogenates) were analysed with the Kruskal–Wallis test followed by Dunn’s multiple comparison post hoc test. A level of p < 0.05 was considered significant.

## 5. Conclusions

In conclusion, our experimental data show that both P-WPS and AF-WPS induced a hypercoagulable state, increased systolic blood pressure, troponin I, oxidative stress and DNA damage in heart homogenates. We also demonstrated that the expression of Nrf2 and oxidative stress markers in the heart were more consistently affected by AF-WPS inhalation. Moreover, markers of inflammation (TNFα and IL1β) and cleaved caspase-3 were only significantly increased in AF-WPS exposure, suggesting that flavouring added to tobacco used in WPS induces more cardiovascular toxicity than unflavoured WPS. Further studies are needed to evaluate the chemical composition of P-WPS and AF-WSP which could be different due to the presence of various ingredients in flavoured WPS tobacco.
